# Extracorporeal Membrane Oxygenation-Impella (ECPELLA) in a Patient With Recalcitrant Ventricular Tachycardia Undergoing Ablation

**DOI:** 10.7759/cureus.38114

**Published:** 2023-04-25

**Authors:** Kripa Rajak, Anupam Halder, Smit Paghdar, Smruti P Desai, Jose Ruiz, Rohan Goswami

**Affiliations:** 1 Internal Medicine, University of Pittsburgh Medical Center (UPMC) Harrisburg, Harrisburg, USA; 2 Transplant, Mayo Clinic, Jacksonville, USA

**Keywords:** impella 5.5, va-ecmo, cardiogenic shock, temporary mechanical circualtory support, ventricular tachycardia (vt) storm

## Abstract

In advanced heart failure, refractory hemodynamically unstable ventricular tachycardia (VT) can be life-threatening. The utilization of short-term temporary mechanical circulatory support (MCS) has been described. Still, it is limited to the intra-aortic balloon pump (IABP) or Impella 2.5/CP (Abiomed Inc., Danvers, MA, USA) which may only provide up to 1-2.5 L/min of added support. Escalation of MCS therapies should be considered. Referrals to advanced tertiary heart transplant centers should be done early to afford patients the best chance at an optimal outcome, with the option for heart transplant evaluation if needed. We present a case of recalcitrant hemodynamically unstable VT complicated by cardiac arrest, eventually undergoing successful VT ablation while supported on veno-arterial extracorporeal membrane oxygenation (VA ECMO) and Impella 5.5 as a vent strategy in the extracorporeal membrane oxygenation-Impella (ECPELLA) configuration.

## Introduction

Refractory hemodynamically unstable ventricular tachycardia (VT) can be life-threatening in patients with cardiogenic shock and should be managed aggressively and early to improve outcomes. Strategies to optimize hemodynamic support before ablation are not well outlined. Currently, no guidelines are available to reference for ablative support [1]. Few case reports demonstrate mechanical circulatory support (MCS) use in adult and pediatric populations [2,3]. Given the increase in heart failure patients in the United States, the likelihood of patients necessitating MCS use to undergo successful ablation is also increasing. We present a case of recalcitrant hemodynamically unstable VT complicated by cardiac arrest, eventually undergoing VT ablation while supported on veno-arterial extracorporeal membrane oxygenation (VA ECMO) and Impella 5.5 as a left ventricular venting strategy in the extracorporeal membrane oxygenation-Impella (ECPELLA) configuration [4].

## Case presentation

**Figure 1:**
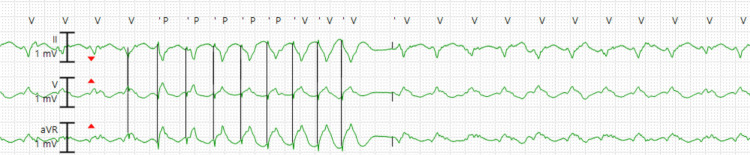
Rhythm strip demonstrating ventricular tachycardia and anti-tachycardia pacing.

**Figure 2:**
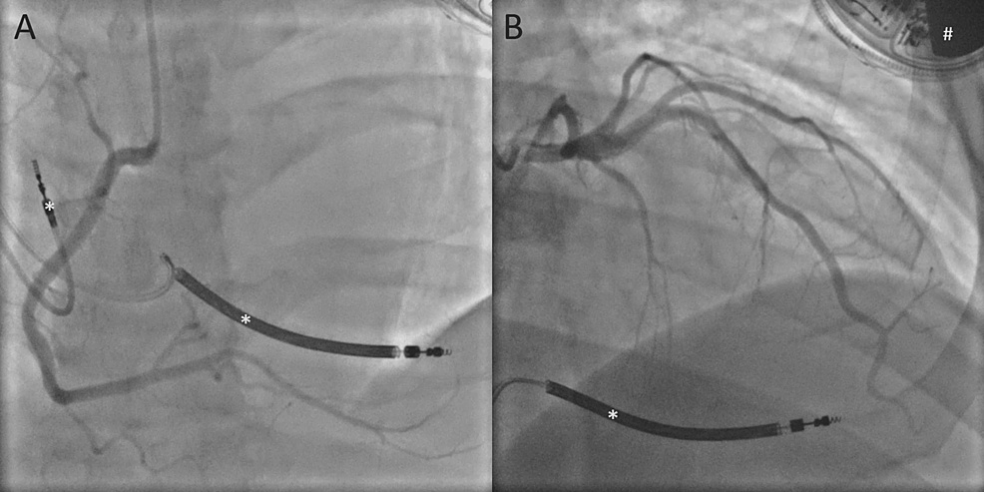
Selective coronary angiography images demonstrating non-obstructive coronary arteries. A: Right coronary artery angiogram; B: left coronary artery angiogram. *: Implantable cardioverter-defibrillator leads in the right atrium and right ventricle (A) and right ventricle (B). #: Implantable cardioverter defibrillator generator (B).

A 63-year-old male with a past medical history of nonischemic cardiomyopathy and a known left ventricular ejection fraction (EF) of 10% status post-dual chamber implantable cardioverter defibrillator (ICD) placement and known VT on oral amiodarone presented with episodes of monomorphic VT requiring internal defibrillation (Figure 1). Initial workup at an outlying facility, including electrolytes and CT head, was unremarkable. Subsequently, to rule out new coronary disease, a left heart catheterization was performed and demonstrated mild luminal irregularities without obstructive coronary artery disease (Figure 2). The patient underwent Impella CP (Abiomed Inc., Danvers, MA, USA)-supported VT ablation. Unfortunately, he had a cardiac arrest during the procedure despite circulatory support and was resuscitated for 90 minutes, intubated, and admitted to the intensive care unit and stabilized. He was then transferred to our facility (on Impella CP support) but required rapid escalation of vasoactive support. 

**Figure 3:**
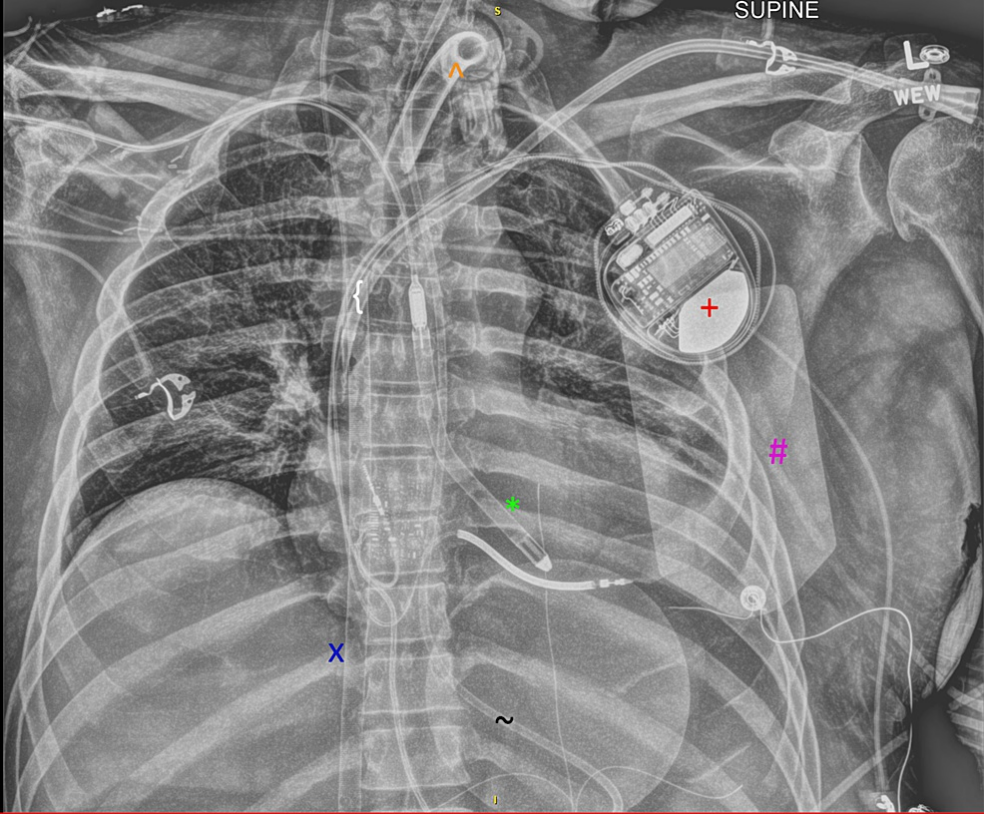
Chest x-ray demonstrating multiple support devices. *: Impella 5.5 in left ventricle, +: implantable cardioverter defibrillator, #: external defibrillator, {: tunneled dialysis catheter, ^: tracheostomy, X: ECMO venous return cannula, ~: nasojejunal feeding tube.

Due to refractory cardiogenic shock and failing to stabilize on Impella CP and multiple vasopressors and inotropes, he was seen by our cardiothoracic surgeon and felt to be a suitable candidate for placement of an axillary Impella 5.5 with smart assist with the hopes for ventilator liberation and rehabilitation. Despite increased medical and mechanical support optimization, the patient had intermittent bouts of sustained VT requiring external defibrillation after failed internal anti-tachycardia pacing and defibrillation, which resulted in circulatory collapse. After multi-disciplinary discussion among cardiothoracic surgery, critical care, electrophysiology, and transplant cardiology teams, the patient underwent placement of VA ECMO with Impella 5.5 remaining as an left ventricular (LV) vent (in the ECPELLA configuration), Figure 3. After stabilization, he successfully underwent a VT ablation with ECPELLA.

Despite optimization, he had progressive organ dysfunction and multi-system organ failure requiring renal replacement therapy. He succumbed to progressive complications, and his family felt it appropriate to withdraw care due to poor neuroprognostication and unresponsiveness when weaned from sedation.

## Discussion

Our case highlights a critical knowledge gap in patients with cardiogenic shock due to recalcitrant ventricular arrhythmias. MCS options that can be used for hemodynamically unstable VT ablation described in the literature are the intra-aortic balloon pump (IABP), Impella 2.5, or CP, which only provide up to 2.5-3.5 L/min of flow. The most clinical experience thus far with VT ablation has been with the Impella 2.5 device [5,6]. Hemodynamic support aims to maintain adequate end-organ perfusion during prolonged episodes of ventricular tachycardia, thereby allowing detailed circuit mapping.

MCS devices have proven their ability to temporize shock, reduce vasoactive support needs, and improve outcomes [7]. However, limited case series and data exist focusing upon temporary support devices such as Impella or a balloon pump. Veno-arterial ECMO has not been sufficiently described within the literature as a bridge to ventricular tachycardia in unstable patients. Miller et al. retrospectively studied 22 patients, comparing VT ablation in the IABP or no mechanical support group with patients supported by Impella 2.5. Impella-supported patients could maintain in VT 2.5-fold longer (66.7 min longer vs. 27.5 min), required fewer premature terminations of the arrhythmia for hemodynamic instability (1.0 vs. 4.0), and had more patients for which at least 1 VT was terminated during ablation (90% vs. 38%) [8]. This study demonstrates the benefit of MCS with Impella 2.5. A multicenter observational study of 66 patients who underwent VT ablation with hemodynamic support-IABP, Impella 2.5, and TandemHeart (Cardiac Assist Inc, Pittsburgh, PA, USA) was used in 22, 25, and 19 patients, respectively [9]. In the MCS group (Impella 2.5 or TandemHeart), more patients underwent entrainment/activation mapping (82% vs. 59%), and more unstable VTs were mapped and ablated per patient (1.05 ± 0.78 vs. 0.32 ± 0.48). More VTs were successfully terminated by ablation (1.59 ± 1.0 vs. 0.91 ± 0.81) [9,10].

Despite this evidence, escalating support for successful VT ablation has not been described, with an incremental stepwise increase from minimal support (e.g., IABP 1 L/min) to maximal support (e.g., VA ECMO 4-8 L/min). Our case is the first to demonstrate that despite 2.5-3 L/min of flow with small devices (IABP or Impella CP), patients with refractory hemodynamically unstable ventricular arrhythmias can benefit from escalating MCS options, such as VA-ECMO or ECPELLA. ECPELLA is an emerging therapy being utilized in both LV offloading and full circulatory support, thereby preserving left ventricular function and reducing LV dilation and wall stress [11]. Utilizing the ECPELLA configuration in our patient provided the added benefit of hemodynamic stability and a reduction in LV afterload while not interfering in our electrophysiological process of circuit mapping, entrainment, or ablation outcome.

As patient longevity increases with newer medical therapy for heart failure (e.g., empagliflozin, sacubitril/valsartan), it is anticipated that more patients with chronic heart failure may present with arrhythmogenic complications of their underlying cardiomyopathy. Further research is needed to highlight the optimal multi-disciplinary strategy for improving patient outcomes by utilizing pre-ablative hemodynamic assessment and planned mechanical circulatory support placement.

## Conclusions

In patients with deranged hemodynamics and refractory shock, it is vital to understand the advanced support options available to the clinician, such as axillary Impella 5.5, VA ECMO, TandemHeart, or ProTek Duo. If needed, escalation of MCS to ECPELLA may improve the procedure’s safety by unloading the left ventricle without affecting the outcome of the ablation, as in our patient. MCS has shown efficacy in maintaining adequate end-organ perfusion during prolonged episodes of VT, thereby offering a longer time for detailed activation and entrainment mapping. Future research must look at the potential benefits of ECPELLA over other devices as a bridge to VT ablation in recalcitrant arrhythmia in order to provide adequate systemic circulatory support in longer-duration ablative procedures beyond that of IABP or Impella CP. The potential advantage of tertiary center care for these complex patients is the availability of resources for urgent heart transplantation evaluation, should it be needed.
